# Fosfomycin Use in Treating Severe Difficult-to-Treat Gram-Negative Infections—A Comprehensive Review

**DOI:** 10.3390/antibiotics15030234

**Published:** 2026-02-24

**Authors:** Despoina Koulenti, Jean-François Timsit

**Affiliations:** 1 Critical Care Department, King’s College Hospital NHS Foundation Trust, London SE5 9RS, UK; deskogr@yahoo.gr; 2 UQ Centre for Clinical Research (UQCCR), The University of Queensland, Brisbane 4072, Australia; 3 Faculty of Medicine, University of Thessaly, 41500 Larissa, Greece; 4 School of Health Sciences, University of Ioannina, 45110 Ioannina, Greece; 5 Medical and Infectious Diseases Intensive Care Unit (MI2), Assistance Publique Hôpitaux de Paris (AP-HP) Bichat Hospital, F-75018 Paris, France; 6 Infection Antimicrobials Modeling Epidemiology (IAME), Institut National de la Recherche Medicale (INSERM), Université Paris-Cité, F-75018 Paris, France; 7 OUTCOME REA Research Network, F-93029 Drancy, France

**Keywords:** sepsis, septic shock, carbapenemases, difficult-to-treat Gram-negative bacteria, extended-spectrum beta-lactamases, fosfomycin, critically ill, pharmacokinetics, metallo-beta-lactamases, repurposed antimicrobials

## Abstract

**Background/Objectives**: Fosfomycin is an old antimicrobial agent historically used in its oral formulation for uncomplicated urinary tract infections. In the current context of rising antimicrobial resistance and limited antimicrobial options, fosfomycin has attracted renewed interest. **Methods:** A comprehensive review on the IV fosfomycin use focusing on critically ill patients and/or severe infections due to difficult-to-treat (DTR) Gram-negative bacilli (GNB). **Results:** Fosfomycin’s IV formulation is now being used more widely, particularly in critically ill patients with multidrug-resistant (MDR) or DTR-GNB infections. It offers several attractive features: a unique mechanism of action that minimizes cross-resistance; a broad spectrum of activity, covering both Gram-negative and Gram-positive pathogens; and consistent synergy with multiple pivotal antimicrobials. Its pharmacokinetic/pharmacodynamic (PK/PD) profile is favorable, with extensive tissue penetration, including the central nervous system. The ratio of area under the concentration–time curve to the minimum inhibitory concentration of the pathogen (AUC/MIC) is considered the optimal PK/PD target for fosfomycin. The adverse events are mainly non-serious (most frequently, hypernatremia and hypokalemia), although safety data for higher dosing regimens remain limited. Growing clinical evidence supports IV fosfomycin as an effective and well-tolerated component of combination therapy for severe infections in critically ill patients, including those infections caused by extended-spectrum beta-lactamases-, carbapenemase-producing Enterobacterales, and DTR non-fermentative GNB. Nevertheless, as with many rediscovered antimicrobials, its expanded role requires confirmation through rigorously designed clinical trials to better define its efficacy, optimal use, and safety profile in the treatment of severe DTR-GNB infections.

## 1. Introduction

An exponential increase in multidrug-resistant microorganisms (MDRs), especially of Gram-negative bacilli (GNB), has been observed during the last decades at a global level [1,2,3,4,5]. The problem of antimicrobial resistance is more prominent in the intensive care unit (ICU) environment due to multiple factors, such as a vulnerable patient population, use of invasive devices, and high antibiotic exposure, resulting in a vicious cycle of further antibiotic use and AMR development [4,6,7]. Strategies to tackle antimicrobial resistance (AMR) include measures to prevent the spread of resistant pathogens; optimization of the use of current antimicrobials, i.e., dosing optimization using pharmacokinetic and pharmacodynamic principles (PK/PD); development of new antimicrobials; and development of alternatives to antimicrobials (e.g., bacteriophages, antimicrobial peptides, antisense therapeutics, microbiota-based therapeutics, antibodies) [8,9,10,11,12]. However, the development of novel antimicrobials involves lengthy research, significant financial investment, and takes a considerable amount of time for antimicrobials to reach the market. In the last decades, there has been a discovery void of novel antimicrobial classes, and the novel branded or in the pipeline antimicrobials are mainly modifications of existing classes or combinations aiming to tackle specific mechanisms of resistance [8,9]. On the other hand, very few alternatives to antimicrobials have reached the stage of clinical trials, and despite the existing potential, they are still far from providing a solution to tackle AMR in the near future [12,13]. Novel antimicrobial development resembles a high-stakes race against microbial resistance evolution, with pathogens, at present, accelerating faster than our ability to innovate.

In view of the current situation of AMR spread and the limited treatment options, re-exploring and re-purposing old antimicrobials, such as fosfomycin, in the battle against AMR, has attracted increasing interest [3,14,15,16]. Fosfomycin has been “rediscovered” due to its in vitro and in vivo activity against a wide range of MDR and extensively drug-resistant (XDR) bacterial strains. Its broad activity against almost all carbapenemase-producing Enterobacterales (CPE), along with the limited efficacy of most novel antimicrobials against metallo-beta-lactamase (MBL) producers, makes fosfomycin an attractive therapeutic choice [3,14,15,16,17,18,19]. An expanding body of evidence indicates that intravenous (IV) fosfomycin is an effective and safe component of combination therapy for a wide spectrum of severe infections in critically ill patients [3,14,15,16,17,18,19,20,21,22]. However, as with all “older” antimicrobials that have been “rediscovered” and “repurposed” in our AMR era, further evidence is needed to establish their new, expanded role as, when they were first branded, they might have not been rigorously studied [23].

The European Medicines Agency (EMA) has approved IV fosfomycin with the indication of treatment of serious infections when other treatments are not suitable, while very recently (22 October 2025), the United States (US) Food and Drug Administration (FDA) approved IV fosfomycin for the treatment of complicated urinary tract infections (cUTIs) in adults, including acute pyelonephritis, caused by susceptible isolates of *Escherichia coli* and *Klebsiella pneumoniae* [24,25,26].

In this comprehensive review, we provide a general description of IV fosfomycin, including mechanism of action, antimicrobial spectrum, and PK/PD characteristics, and we focus on its current role as part of salvage combination regimens for infections caused by difficult-to-treat Gram-negative pathogens, presenting recent clinical trial results, real-world data, and summarizing recommendations based on existing evidence.

## 2. General Description

The molecule. Fosfomycin (*cis*-1,2-epoxypropyl phosphonic acid; C3H7O4P) was discovered in the late 1960s, produced from cultures of *Streptomyces* species [14,15,16,17,18,19]. Currently, it is produced by laboratory synthesis [14,15]. Fosfomycin is the sole commercially available antimicrobial of the phosphonic acid class [14,15,16,17]. It was initially marketed for oral administration as fosfomycin calcium; still, due to better bioavailability, fosfomycin tromethamine has become the standard [14]. An IV formulation of fosfomycin disodium was later made available [14].

**Figure 1:**
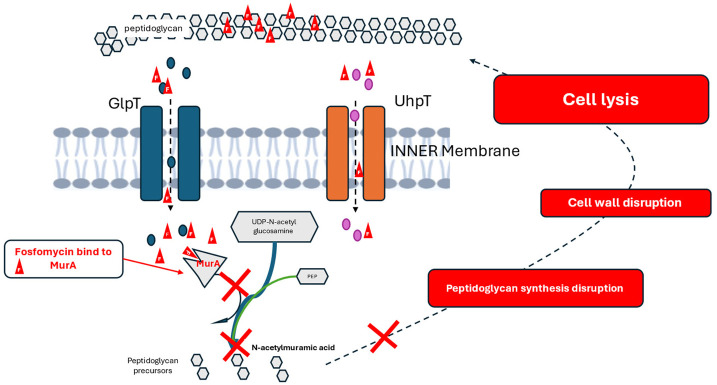
Schematic depiction of the main mechanism of antibacterial activity of fosfomycin. Fosfomycin (symbolized as red triangles) inhibits the first step of bacterial wall synthesis. Following exposure to fosfomycin, it is actively transported into the bacterial cell via the GlpT and UhpT bacterial transporters usually designed to transport Glycerol 3-Phosphate (blue circles) and Glycerol 6-Phosphate (pink circles). Once inside the cell, fosfomycin acts as a structural mimic of PEP and binds to the MurA enzyme, thereby irreversibly blocking (red “x” mark) the condensation of UDP-N-acetylglucosamine with PEP preventing the formation of N-acetylmuramic acid (early peptidoglycan precursor), thus disrupting peptidoglycan synthesis and consequently disrupting bacterial cell wall synthesis and leading to cell lysis and cell death. Image created with BioRender.com. Abbreviations: GlpT: glycerol-3-phosphate transporter, UhpT: hexose-phosphate transporter, F: fosfomycin, MurA: UDP-N-acetylglucosamine enolpyruvyl transferase, PEP: phosphoenolpyruvate, UDP-N-acetylglucosamine: uridine diphosphate-N-acetylglucosamine.

*Mechanism of action.* Fosfomycin is a bactericidal antimicrobial with a unique mechanism of action, i.e., irreversible inhibition of the first step of bacterial wall synthesis [14,15,16,17,18,19]. The target of fosfomycin is the phosphoenolpyruvate transferase (MurA), a crucial enzyme that catalyzes the first step of peptidoglycan biosynthesis [14,15,16,17,18,19]. Once fosfomycin enters the bacterial cytoplasm, it binds irreversibly to a cysteine residue in the active site of MurA, preventing it from catalyzing the formation of peptidoglycan precursor UDP-N-acetylmuranic acid, leading to bacterial wall disruption and consequently to cell lysis and bacterial death (Figure 1) [14,15,16,17,18,19]. Another mechanism of action of fosfomycin is the decrease in penicillin-binding proteins [13]. These unique mechanisms of action of fosfomycin allow for synergy with other antimicrobials, such as beta-lactams, aminoglycosides, fluoroquinolones, and oxazolidinones [14,15,16,17,18,19,27,28,29,30]. This in vitro synergy has also been documented in DTR Enterobacterales and *Pseudomonas aeruginosa* [31,32].

Interestingly, apart from the above-described antibacterial properties, fosfomycin has other actions, i.e., anti-inflammatory and immunomodulatory ones, such as alteration of the function of leukocytes, particularly of neutrophils [33]. Specifically, it has been shown that incubation of neutrophils with fosfomycin leads to enhancement of their bactericidal ability, to an increase in their intracellular calcium concentrations, to an elevation of extracellular reactive oxygen intermediate production, and to a decrease in chemotaxis, without affecting chemokinesis [33]. In addition, it has been reported that in in vitro experimental models, fosfomycin affects immunoglobulin production by B cells, suppresses T cells, and affects cytokine production by T cells, while it also promotes tissue regeneration through enhancement of collagen deposition, angiogenesis, and wound healing [34,35,36,37,38].

## 3. Antimicrobial Spectrum

Fosfomycin has a wide spectrum of activity, including Gram-positive and Gram-negative microorganisms [14,15,16,17,18,19,39]. The Gram-positive activity of fosfomycin also includes resistant pathogens, such as methicillin-resistant *Staphylococcus aureus* (MRSA) and vancomycin-resistant *Enterococcus* (VRE) [14,15]. The activity of fosfomycin against GNB includes most of the CPEs, such as *E. coli*, *K. pneumoniae*, *Proteus mirabilis*, *Proteus vulgaris*, *Citrobacter species*, and others with low minimum inhibitory concentrations (MIC) ranging from 0.25 to 16 mg/L; however, for several of these species, acquired resistance might be a problem, with observed MICs of 64 mg/L (European Committee on Antimicrobial susceptibility testing (EUCAST) threshold of resistance 32 mg/L) [17,39]. *P. aeruginosa* and *Acinetobacter baumannii* show moderate susceptibility to fosfomycin (16–64 mg/L), but activity in MDR strains is unpredictable and varies across phenotypes [17,39]. Several *Acinetobacter* species are also not susceptible [17]. Organisms inherently resistant to fosfomycin include the Gram-positive *Staphylococcus saprophyticus* and *Streptococcus pyogenes*, the aerobic Gram-negative *Morganella morganii* (Enterobacterales), the non-fermenter *Stenotrophomonas maltophilia*, *Legionella pneumophila*, *Bacteroides* spp. (anaerobes), and the atypical pathogens *Chlamydia* spp., *Chlamydophila* spp., and *Mycoplasma* spp. [39].

Regarding MDR microorganisms, fosfomycin has shown in numerous studies in vitro and in vivo activity against MDR and XDR strains of GNB [14,15,17,19,20,21,40]. It has good activity against Enterobacterales producing extended-spectrum beta-lactamases (ESBLs), although MIC90 may differ between regions [14,15,16,17,20,21,41]. Fosfomycin has activity against CPEs, including MBLs [3,14,15,16,17,20,21,41,42]. There are reports in which fosfomycin was active against > 90% of MBL-producing Enterobacterales; however, its activity varies significantly, and it is also difficult to compare the results of different studies due to the heterogeneity of the susceptibility testing methods and different MIC values used [3,14,41,42,43,44,45].

It should be kept in mind that neither the disc diffusion method nor the broth microdilution method provides truly correct results of fosfomycin susceptibility testing. Both the EUCAST and Clinical Laboratory Standards Institute (CLSI) recommend agar dilution as a gold standard method for this purpose [46,47]. Due to these uncertainties, known breakpoints for fosfomycin have been largely reduced by the EUCAST [46].

Moreover, due to baseline heteroresistance and frequent increase in measured MIC under fosfomycin treatment, it is recommended not to use fosfomycin monotherapy against most of the severe bacterial infections, including MBL-producing Enterobacterales [3]. Against strains of *K. pneumoniae* producing New Delhi MBLs (NDA), fosfomycin displays synergy with carbapenems and/or colistin [3]. The activity of fosfomycin against carbapenem-resistant *P. aeruginosa* (CRPA) is variable as well [41]. Table 1 summarizes the potential in vitro activity of fosfomycin against carbapenem-resistant GNB [41].

## 4. Resistance

Resistance to fosfomycin can be inherent or acquired and may occur via chromosomal mutations or may be plasmid borne [48]. The major mechanisms of resistance development are via spontaneous chromosomal mutations that lead to a reduction in bacterial permeability to fosfomycin (leading to decreased uptake from the bacterial cells) or modifications of the MurA target [14,15,17,39]. Resistance mechanisms via plasmids lead to hydrolytic enzymatic inactivation of fosfomycin [48]. It is noteworthy that due to the unique, one-step mechanism of action of fosfomycin, no cross-resistance with other antibacterial classes is known [14,15,17,39]. Although resistance has been found to develop rapidly in vitro, in vivo development appears to be slower [14,15,17,39]. However, during monotherapy with fosfomycin, there are reports of rapid resistance development for ESBL-producing *E. coli*, *P. aeruginosa*, *K. pneumoniae*, VRE, and CRE, with consequent advocacy for fosfomycin to be used in combination for the treatment of MDR pathogens [14,15,17,39]. Moreover, mechanisms of resistance to fosfomycin may differ between *P. aeruginosa* and Enterobacterales, a fact that might explain the higher MICs of *Pseudomonas*; consequently, *E. coli* MIC breakpoints should not be extrapolated to *Pseudomonas* [49].

## 5. PK/PD Characteristics

Fosfomycin is an antimicrobial with very low molecular weight (138.059 g/mol), hydrophilic, with low volume of distribution (Vd 0.4–0.5 L/kg; distributes exclusively in the extracellular fluid), not metabolized, and is mainly renally cleared (70–80% excreted unchanged in the urine); the elimination half-life of IV fosfomycin in normal renal function is 2.8+/−0.6 h (in renal impairment the half-life of fosfomycin is increased proportionally to the degree of impairment) [14,15,17,18,19,39,48]. Due to its low Vd and renal clearance, fosfomycin levels are significantly affected by increased Vd, augmented renal clearance, or impaired renal function, which are very common in critical illness, and significant PK variability has been reported in the critically ill population [18,19]. The protein binding of fosfomycin is negligible (<5%), so its PKs are unaffected by albumin levels [17,18,19,50]. The oral formulation, fosfomycin tromethamine, has good bioavailability, but IV administration of fosfomycin achieves much higher peak concentrations [14]. Fosfomycin also shows excellent tissue penetration, including the central nervous system (CNS) [14,15,17,51,52]. Specifically, fosfomycin has a 50–70% penetration into the cerebrospinal fluid when the meninges are strongly inflamed (27% penetration when non- or moderately inflamed meninges), 30–50% into lungs, and 40% into subcutaneous tissues, while penetration in the bones and to the aqueous humor is lower: 20% and 15%, respectively [17,39]. One recent study detailed the lung penetration in 37 healthy volunteers and showed that after the third dose of 6 g (every 8 h) administration, a concentration in the epithelium lining fluid of 15.7 to 82 µg/mL (penetration of 30.8%) and in the alveolar macrophages of 14.8 to 32 µg/mL (penetration of 16.8%) [53]. The accompanying PK/PD model suggest that ELF concentration will be sufficient for *P. aeruginosa* with MIC of 16 mg/L. However, a dose as high as 8 g every 8 h will be needed for strains with an MIC of 32–64 mg/L [54]. Other notable and clinically relevant characteristics of fosfomycin are the biofilm penetration and the capacity for intracellular bactericidal killing [17,55].

**PK/PD targets.** The PK profile of fosfomycin is a predictable, dose-proportional pharmacokinetic profile [39]. Regarding the best PK/PD index predicting efficacy, initial studies had suggested that the time of free drug concentration above the MIC (fT/MIC) was the optimal index [15]. However, further studies showed that the PK/PD index better associated with fosfomycin’s efficacy is the ratio of the area under the free drug concentration–time curve and MIC of a pathogen (fAUC/MIC) [15,48,56,57]. For Enterobacterales, the suggested target for bactericidal activity is a ratio of AUC from 24–48 h and MIC that exceeds 83.3 (AUC_24–48_/MIC ratio > 83.3) and, at the same time, percentage of time above MIC that exceeds 69.0 (fT_24–48_/MIC > 69) [58]. For Pseudomonas, 1-log kill has been observed for AUC/MIC exceeding 15.6 or 40.8; however, other studies have shown the need for higher values; in an in vitro model, for suppression of resistance development, an AUC/MIC ratio of 489 to 1024 was required, although these targets were impossible to be achieved within the safe clinical dosing range [57,59].

## 6. Dosing

Effective dosing is crucial for achieving target attainment and optimal outcomes, particularly for critically ill patients with MDR infections [15,18,19,57]. Although PK/PD targets for effectiveness have been suggested (see PK/PD section), the dosing regimens of fosfomycin for maximal activity with the least toxicity have not yet been fully characterized, i.e., doses that maximize efficacy and, at the same time, minimize adverse events, particularly in presence of renal impairment [57].

In the US, in the product prescribing information, the recommended dose of IV fosfomycin for patients with estimated creatinine clearance (CrCl) > 50 mL/min is 6 g administered every 8 h over 1 h infusion, and dosage adjustments are recommended in case of CrCl < 50 mL/L (see Table 2); the suggested treatment duration is up to 14 days, guided by the infection severity and the assessment of patient’s clinical status [48]. On the other hand, the dosage recommendation of the IV fosfomycin approved in Europe, for patients with CrCl ≥ 80 mL/min, ranges from 12 to 24 g/day depending on the type of infection (24 g for CNS focus of infection or MDR pathogens), divided in 2 to 3 doses (maximum 8 g/dose), with recommended dosage adjustments (percentage decreases) for CrCl < 40 mL/min (Table 2) [24,25,39,60,61]. It is noteworthy that for CrCl between 40 and 80 mL/min, although in the prescribing information, no dosage adjustment is recommended; EMA advises to exercise caution if administered doses are at the higher end of the recommended range [25,60]. This caution is necessary because the optimal dosing within the 12 to 24 g range/day has not yet been clearly established for this renal function group, particularly at the higher end of the dosing spectrum, where the balance between efficacy and toxicity is more challenging; similar optimal dosing uncertainty exists for patients undergoing renal replacement therapy (RRT), as fosfomycin’s clearance may be affected by several factors, such as type of RRT, modality, and settings [56,57]. Nevertheless, more data are increasingly becoming available and progressively improving our understanding of fosfomycin exposure–response relationships in these patient groups [56,57].

A recent study by Wangchinda et al. used a population PK one-compartment model (Monte Carlo simulations of dosing regimens) to determine the optimal dose of fosfomycin for treating MDR GNB infections, while minimizing adverse effects, taking into consideration the varying renal function of the patients [57]. Fosfomycin’s clearance was affected by CrCl and gender [57]. The PK-model proposed a renally adjusted dosing using the lowest dose able to achieve ≥90% probability of target attainment (PTA) according to the EUCAST breakpoints [57]. It was proposed that in patients with normal renal function (estimated CrCl 91–120 mL/min), a dose of 15 g/day was effective against *E. coli* and *K. pneumoniae* with MIC ≤32 mg/L; however, it was noted that for *P. aeruginosa*, higher doses might be needed [57]. Interestingly, for renal impairment, the suggested doses of the above model were generally lower than those used in clinical practice, a fact that the authors highlighted to be considered as it may improve tolerability [57].

Finally, another very recent population PK study by Götz et al. explored optimal dosing of IV fosfomycin for critically ill patients with or without RRT using data from four prospective, observational cohorts; the included data were from critically ill patients without RRT, with prolonged-intermittent RRT (PIRRT) or continuous RRT (CRRT) [56]. The aim of the study was to combine the heterogenous datasets and develop a comprehensive PK model accounting for the RRT that would enable the establishment of optimized fosfomycin dosing regimens for a variety of critically ill patients’ scenarios [56]. Compared to the study by Wangchinda, a two-compartment model (body clearance and dialysis clearance) was used, along with more sampling points [56,57]. In the final model, the eGFR_MDRD_ was implemented and, to minimize potential overestimation of individual renal function resulting from the use of a creatinine-based equation, body clearance was excluded for anuric patients [56]. The dataset included a wide range of renal function, from normal (eGFR_MDRD_ < 30 mL/min/1.73 m^2^) to severely impaired (eGFR_MDRD_ ≥ 90 mL/min/1.73 m^2^). For the subgroup of patients with acute kidney injury on RRT, significant covariates affecting fosfomycin’s PKs were the estimated glomerular filtration rate (eGFR), the dialysate flow rate, and the timing after the first dose of fosfomycin to sampling [56]. In terms of optimal dosing, the study concluded that for critically ill patients with severely impaired renal function during CRRT or PIRRT (eGFR_MDRD_ ≤ 30 mL/min/1.73 m^2^) or without RRT (eGFR_MDRD_ ≤ 60 mL/min/1.73 m^2^), a dose of 5 g every 8 h may be appropriate for bactericidal activity for MIC= 32 mg/L [56]. On the other hand, for critically ill patients with improving renal function during RRT or without RRT (eGFR_MDRD_ ≤ 90 mL/min/1.73 m^2^), a dose of 8 g every 8 h may need to be considered for treatment optimization against MDR pathogens (Table 2) [56]. It should be noted that for MIC = 64 mg/L, even dosage of 8 g/8 h is mainly bacteriostatic, while for MIC = 124 mg/L, it is only bacteriostatic [56].

## 7. Therapeutic Drug Monitoring (TDM)

TDM is not widely used for fosfomycin. A single-center retrospective study assessed the adverse events of IV fosfomycin use in a cohort of 224 patients and, in a subgroup of 68 (30.4%) patients for whom TDM data were available, explored the role of TDM in predicting them [62]. From the cohort, 36.2% were ICU patients, 15.7% had septic shock, and the most frequent infection site was the lower respiratory tract (55.4%) [62]. Trough levels (minimum concentration—Cmin) and steady state levels (Css) were assessed for intermittent (40 patients) and continuous (38 patients) fosfomycin IV infusions, respectively, to evaluate adverse events predictions in ≤5 days from measurement [62]. Overall, the findings support previous data corroborating the good safety profile; fosfomycin treatment was discontinued in 17% of the patient cohort, but this was related to the underlying disease severity and not the use of fosfomycin [62]. At least one adverse event was experienced by 42.4% of the cohort, with a median time of 4 days (interquartile range [IQR] 2–7 days) from fosfomycin initiation; however, adverse events were mostly non-serious [62]. The most frequent adverse event was hypernatremia (23.7%), followed by hypokalemia (23.2%), diarrhea (21.1%), nausea (12.6%), and transaminases increase (12.6%) [62]. Risk factors for adverse events included ICU admission, presence of septic shock, and focus of infection in the lower respiratory tract, while the total daily dose of fosfomycin was not related [62]. The median time that TDM samples were obtained was after 3.5 days (2.5–6.0) from fosfomycin’s initiation; the median value of Cmin was 171.5 mg/L (68.5–244.5), while the median value of Css was 188.8 mg/L (138.0–329.0) [62]. The TDM values predicted the development of adverse events overall with an area under the receiver operating characteristic curve (AUROC) of 0.65 (95%CI:0.44–0.86) and 0.67 (95%CI:0.390.95) for Cmin and Css, respectively, and the development of hypernatremia with an AUROC of 0.91 (95%CI:0.79–1.0) and 0.76 (95%CI:0.52–1.0) for Cmin and Css, respectively [62]. Although the number of patients with TDM performed was small (TDM was requested on a case-by-case basis only, usually ≥48 h from fosfomycin initiation), there is a signal of a potential role of TDM to predict fosfomycin-related adverse events close to TDM assessment that should be explored in further studies [62].

## 8. Clinical Trials and Real-World Data

The FOREST randomized controlled trial (RCT) investigated whether IV fosfomycin is non-inferior to ceftriaxone or meropenem (if bacteria resistant to ceftriaxone) in the targeted treatment of bacteremic UTIs caused by MDR *E. coli* (143 participants) [20]. Non-inferiority was not demonstrated because of a high discontinuation rate of fosfomycin due to adverse events (6 patients with heart failure (8.5%) in the fosfomycin arm vs. 0 patients in the comparator arm, *p* = 0.006) [20]. Interestingly, among patients completing treatment and assessed at test-of-cure, clinical failure occurred in 14.3% vs. 19.7% (risk difference −5.4 percentage points; 1-sided 95% CI −∞ to 4.9) [20]. It was claimed that fosfomycin might be an option in selected patients with bacteremic UTIs [20]. Furthermore, exploratory analysis showed lower new rectal colonization with ceftriaxone- or meropenem-resistant GNB in the fosfomycin arm [20]. A post hoc DOOR analysis suggested the highest probability of better outcomes with fosfomycin was in the clinically evaluable patients and in those without chronic heart disease or renal insufficiency [63].

The ZEUS RCT compared IV fosfomycin (6 g intermittent infusion every 8 h) to piperacillin-tazobactam in hospitalized patients with suspected or microbiologically confirmed cUTI, including pyelonephritis; 465 patients were randomized (<10% bacteremic) [64]. In the microbiologic modified intent-to-treat population (362 patients), IV fosfomycin was non-inferior (success 64.7% vs. 54.5%) [62]. These results supported FDA approval of IV fosfomycin [48,64].

In both FOREST and ZEUS RCTs, subgroup analyses of cephalosporin-resistant or ESBL-producing Enterobacterales showed no significant differences in clinical or microbiological cure; however, these studies were not designed to provide convincing data for critically ill patients, neither for DTR-GNB infections [20,64].

One double-blind, multicenter, investigator-driven, phase 3 RCT comparing monotherapy with ceftazidime or ceftazidime-avibactam vs. combination with IV fosfomycin in adult patients with suspected severe Gram-negative bacterial infections (CAVIFOS trial -NCT07063095) is ongoing [65]. In the multinational platform trial TREAT-GNB (NCT07004049), an intervention comparing ceftazidime/avibactam alone vs. in combination with IV fosfomycin for carbapenem-resistant *P. aeruginosa* and CPE infections is planned to be conducted in Malaysia, Thailand, and Singapore [66].

A German–Austrian prospective, multicenter study assessed real-life use of IV fosfomycin in 209 patients (93% in the ICU) receiving 13.7 ± 3.5 g of IV fosfomycin for 12.4 ± 8.6 days (99% as part of combination antimicrobial treatment) [67]. The most common infections were CNS infections (21.5%), community-acquired pneumonia, hospital-acquired-pneumonia (HAP) and ventilator-associated pneumonia (VAP) (15.3%), bone and joint infections (11%), intra-abdominal infections (11%), and bacteremia (10.5%), while the most frequently identified pathogens were *S. aureus* (22.3%), *S. epidermidis* (14.2%), *Enterococcus* spp. (10.8%), *E. coli* (12.3%), and *Klebsiella* spp. (7.7%); in 24.4% of the cases, ≥1 MDR pathogen was identified [65]. Clinical success was 81.3% overall and 84.8% in MDR cases [67].

The FORTRESS European real-world study aimed to evaluate IV fosfomycin in 1500 patients [21]. Interim results included 718 patients, 51.7% were ICU patients, the mean APACHE II score was 18.3, 5.9% had septic shock, and the overall in-hospital mortality was 10.6% [21]. IV fosfomycin was part of a combination regimen in 90.2% and targeted therapy in 54.5% [21]. The median daily dose was 15 g/day, and the mean duration of fosfomycin treatment was 13.6 days (range 8.1 days to 18.1 days, depending on the indication) [21]. The estimated mean time from the suspected diagnosis of infection to the IV fosfomycin initiation was 5.7 days [21]. The most frequent indications for IV fosfomycin use were bacteremia/sepsis (23.6%), cUTIs (18.0%), and bone and joint infections (17.4%), followed by HAP/VAP (11.0%), complicated skin and soft tissue infections (9.1%), CNS infections (7.8%), and infective endocarditis (6.4%) [21]. The most frequent pathogens were *S. aureus* (31.4%), including MRSA; *Klebsiella* spp. (17.2%), including *K. pneumoniae*; *E. coli* (14.2%); coagulase-negative staphylococci (12.9%); other Enterobacterales (10.9%); and *P. aeruginosa* (8.4%) [21]. MDR pathogens were present in 34.6% of the cases; carbapenem resistance in *Klebsiella* spp. reached 48% [21]. Overall, the clinical success at end-of-cure (primary endpoint) was 75.3%, while the microbiological cure was 82.4% [21]. The clinical success overall for the MDR pathogens was 78%, whereas for the subgroup of carbapenem-resistant GNB, it was 81.8% [21]. Based on the study results, the authors suggested IV fosfomycin as an effective and safe component of combination regimens for severe infections caused by a wide range of Gram-positive and Gram-negative pathogens, including deep-seated and MDR cases [21].

Abdallah et al. reported a retrospective cohort of 30 patients with DTR-GNB infections, 57% UTI, 13% bloodstream infections (BSIs) treated with IV fosfomycin (median 18 g/day (IQR 6–24)); 76% received combination therapy [68]. Carbapenem-resistant *K. pneumoniae* and *E. coli* predominated. Bacterial eradication occurred in two-thirds; 7/30 (23%) died within 30 days, 4 with uncontrolled infection [68].

Another inverse-probability-of-treatment weighting (IPTW) adjusted retrospective trial studied the benefit of combination with IV Fosfomycin in 363 episodes of Gram-negative BSIs [69]. At GNB-BSI onset, the median SOFA score was 5 (2–7), and 122 patients (34%) presented with septic shock [69]. Pathogens were principally *K. pneumoniae* (42%), *E. coli* (28%), and *P. aeruginosa* (17%); of them, 36% were carbapenem-resistant [69]. Fosfomycin MICs was tested in 134 cases, resulting in a median (IQR) MIC of 32 mg/L (16–32) in 92% of the cases [69]. For the 12 remaining strains (3 MDR *P. aeruginosa* and 9 carbapenem-resistant *Klebsiella*), the median MIC was 256 mg/L) [69]. A combination with IV fosfomycin was used in 98 (27%) cases at a median dosage of 16 (16–18) g/daily [69]. The crude 14-day mortality (9% vs. 20%), microbiological eradication (90% vs. 79%), and treatment failure (18% vs. 29%) were significantly better with combinations that included IV fosfomycin [69]. Adverse events leading to treatment discontinuation were higher with fosfomycin (12% vs. 5%, *p* = 0.042) [69]. The IPTW adjusted hazard of 30-day mortality was in favor of fosfomycin use (hazard ratio (HR) = 0.53, *p* = 0.022) [69].

Another retrospective cohort study included 134 patients (ICU 39%, sepsis or septic shock 66%) with carbapenem-resistant Enterobacterales BSI (*K. pneumoniae* 86%, *E. coli* 11.9%, *Enterobacter* 2.1%) that exhibited mostly *bla*NDM-1, *bla*OXA-48, or both [68]. A fosfomycin-based combination was used in 23 cases [70]. On a multivariate analysis adjusted on age, neutropenia, and occurrence of sepsis, the fosfomycin-based regimen was a protective factor of 14-day mortality [70].

Regarding the role of fosfomycin as part of combination treatment for *A. baumannii*, a prospective, multicenter, observational study assessed in real-life conditions the efficacy of fosfomycin-containing antimicrobial regimens in the HAP, including VAP caused by CRAB strains [71]. Among 180 patients (79% ICU), 157 monomicrobial cases were analyzed; 44 received IV fosfomycin in combination as definitive therapy [71]. Septic shock developed in 67.7% of the patients, and the 30-day mortality was 56.1% [71]. *A. baumannii* isolates were 97.6% XDR and 2.4% pandrug-resistant [71]. In the Cox regression analysis, after propensity score adjustment, administration of a fosfomycin-containing regimen was associated with a better 30-day survival [67]. In this study, involving only 2 hospitals in Italy, the MICs of *A. baumannii* to fosfomycin were not provided, questioning the external applicability of these encouraging results [71].

The same group published a retrospective multicenter cohort of 102 patients (90% ICU) with severe CRAB infections (VAP 59%, BSI 22%, catheter-related infection 16%) who received IV fosfomycin in combination mainly with cefiderocol (n = 54), colistin (n = 48), or ampicillin/sulbactam (n = 18) [72]. Clinical failure was 58/102 (57%), and mortality was 48/102 (47%) [70]. In adjusted analyses, colistin-based regimens were associated with higher 30-day mortality, whereas cefiderocol-based regimens were associated with lower 30-day mortality [72].

A small case series of 20 ICU patients in one Greek hospital with BSIs due to CRAB also resistant to colistin and with a MIC to tigecycline > 2 mg/L was reported [73]. In this study, combination rescue therapies using meropenem, colistin, cyclins, aminoglycosides, cotrimoxazole, or fosfomycin (cefiderocol and eravacycline were not available) were administered [73]. Survival rate was 50%, and bacterial eradication was obtained in 14 cases [73]. Fosfomycin-containing regimens were administered in 8 cases and were associated with a better survival (7/8, *p* = 0.02) and a 100% bacterial eradication [73].

A single-center open-label RCT comparing colistin monotherapy with colistin combined with fosfomycin for CRAB with MICs to colistin > 2 mg/L is ongoing in Thailand (NCT06570850) [74]. Finally, another single-center phase 4 (TheraCRAB) study involving ICU patients with CRAB infection randomized to receive IV colistin with either sulbactam or eravacycline or IV fosfomycin is also ongoing (NCT06440304) [75].

## 9. Adverse Events

The most frequent adverse events of fosfomycin, i.e., incidence ≥ 2%, include electrolyte disorders (hypernatremia, hypokalemia, hypocalcemia, hypophosphatemia), transaminase elevations, nausea, vomiting, diarrhea, headache, and neutropenia [48]. Hypokalemia results from increased potassium urinary secretion and is reversible after discontinuation of fosfomycin [15]. Fosfomycin has high sodium content, i.e., 1 g of IV fosfomycin (equivalent of 1.32 g fosfomycin disodium) contains 0.32 g (14 mmol) of sodium (corresponding to the 16% of the recommended maximum daily intake), and hypernatremia risk should be carefully evaluated in patients with heart failure, impaired renal function, hypertension, hyperaldosteronism, and also, critically ill patients with head injury [15,60].

In the FOREST RCT, 8.6% of the patients in the IV fosfomycin arm developed heart failure compared to 1.4% in the meropenem arm, and caution was advised for fosfomycin use in patients with heart failure [20]. In the ZEUS RCT, at least one adverse event was experienced by 42.1% and 32% of the patients in the IV fosfomycin and piperacillin/tazobactam arm, respectively [64]. However, there was no death in the trial, and most of the adverse events were mild and transient (asymptomatic laboratory abnormalities and gastrointestinal events); serious adverse events were uncommon (2.1% and 2.6% for fosfomycin and piperacillin/tazobactam, respectively) [64].

The FORTRESS study reported 8.2% and 33.9% serious and non-serious adverse reactions, respectively [21]. Regarding electrolyte disorders, hypokalemia was recorded in 26.4% of the patients, it was severe in 6.9% and moderate in 41.8% and led to fosfomycin withdrawal in 5.8% of the cases of hypokalemia [21]. Hypernatremia was recorded in 15.2% of the patients, it was severe in 3.7% and moderate in 22% and led to fosfomycin withdrawal in 11% of the cases of hypernatremia [21]. A recent Italian retrospective, real-world, single-center study that assessed the adverse events of IV fosfomycin (11 days of mean duration of treatment) reported an overall good safety profile, as the adverse events, although frequent (42.4% ≥ 1 adverse event), were generally non-serious and led to discontinuation of treatment in less than 20% of cases [60]. Hypernatremia (23.7%) and hypokalemia (9.8%) were the most frequently reported adverse events [62]. Finally, the German–Austrian real-world prospective, multicenter study reported that 16.3% of the patients developed one or more adverse events; however, in most cases, they were non-serious events (only 1.4% serious) [67].

Regarding prescribing warnings for IV fosfomycin due to potential QT interval prolongation in some patients, it is recommended to be avoided in patients with known QT prolongation, ventricular arrhythmias, or torsade de pointes history and to be avoided when co-administered with other medications that prolong the QT (for the full list of warnings and precautions, we refer the reader to the product prescribing information) [48].

## 10. Guideline Recommendations

The ESCMID 2022 guidelines on management of MDR GNB are presented in Table 3 [41]. They provide a strong recommendation for the use of IV fosfomycin in patients with cUTI without septic shock and a conditional recommendation for use in combination with other in vitro active antimicrobials for the treatment of CRE or CRPA when other options are unavailable [41]. For IV fosfomycin monotherapy treatment for CREs, ESCMID 2022 guidelines do not provide any recommendation; it states “potential efficacy of IV fosfomycin for CRE reported in in vitro studies/small case series; however, no clear evidence exists” [41].

The Infectious Diseases Society of America (IDSA) 2024 guidance for the treatment of infections by MDR GNB states that fosfomycin is not suggested as a component of combination treatment for infections from CRAB [76]. Moreover, IDSA guidelines do not suggest fosfomycin for pyelonephritis or cUTI treatment; however, the guidelines were released before the 2025 approval of IV fosfomycin by the FDA for this indication [26,48,76].

The experts from Italian and French infectious diseases societies (Italian Society of Infectious and Tropical Diseases and the French Society of Infectious Diseases) also suggested the use of fosfomycin in combination with ceftazidime-avibactam in CNS infections due to DTR *P. aeruginosa* [77]. They also proposed to combine cefiderocol or the most active beta-lactam/beta-lactamase inhibitor with fosfomycin in case of lower respiratory tract infections due to DTR *P. aeruginosa* not susceptible to ceftolozane-tazobactam [76].

## 11. Purposing Fosfomycin in the Treatment of DTR Gram-Negative Infections in Critically Ill Patients

Managing DTR-GNBs in critically ill patients remains highly challenging. Even among carbapenemase-producing Enterobacterales, for which new agents now provide effective options against Ambler class A and D enzymes, the treatment of Ambler class B MBL-producers remains problematic, with very limited therapeutic alternatives. Similarly, for DTR *P. aeruginosa* resistant to newer backbone agents, such as, ceftolozane-tazobactam, the available options are scarce.

In this context, antibiotics with excellent tissue penetration and synergistic potential with existing backbone therapies may play a meaningful role, particularly in situations with high bacterial inoculum (e.g., VAP) or limited drug diffusion (e.g., CNS infections). CRAB represents another major concern, given its high associated mortality, the paucity of effective new agents, and the increasing resistance to colistin and tigecycline.

Based on our comprehensive review of the available literature, we discovered the following:

**1. IV fosfomycin offers several important advantages:**

**Very broad antibacterial spectrum**, covering susceptible and resistant Enterobacterales and *P. aeruginosa*, as well as Gram-positive pathogens, including MRSA and *Enterococcus* spp.

**Excellent tissue penetration**, notably into the CNS.

**A unique mechanism of action**, associated with a low risk of cross-resistance with other antibiotic classes.

**Strong synergistic activity** when combined with several key antibiotic families.

**Well-characterized pharmacokinetics**, allowing doses up to 24 g/day (in patients with normal renal function) to be used in the ICU setting. At these doses, the probability of achieving bactericidal targets exceeds 90% for isolates with MIC ≤ 32 mg/L.

**Acceptable safety profile** and encouraging clinical outcomes in severe DTR-GNB infections, suggesting that combining IV fosfomycin with other active agents may enhance clinical success and bacterial eradication.

**2. The main limitations of the molecule include the following:**

**Adverse effects related to sodium load**, which increase with higher dosing. Careful dose optimization is essential, particularly given the marked fluctuations in renal function and the frequent use of renal replacement therapy in ICU patients.

**Lack of standardized breakpoints** for many DTR-GNB, combined with difficulties in accurately determining fosfomycin’s MICs and the risk of heteroresistance. In retrospective studies suggesting benefit from fosfomycin-based combinations, MICs were often unreported or ≤32 mg/L.

**Frequent need for high dosing** when MICs are 16–32 mg/L or higher to optimize PTA. After the loading dose, maintenance dosing must be carefully adjusted to renal function.

**Likely requirement for combination therapy** in the most severely ill patients, as monotherapy is unlikely to be sufficient

**Scarcity of well-designed prospective trials** evaluating fosfomycin in severe DTR-GNB infections.

**Limited prospective data in critically ill populations**, with most available evidence derived from retrospective case series subject to information and publication biases.

**Figure 2:**
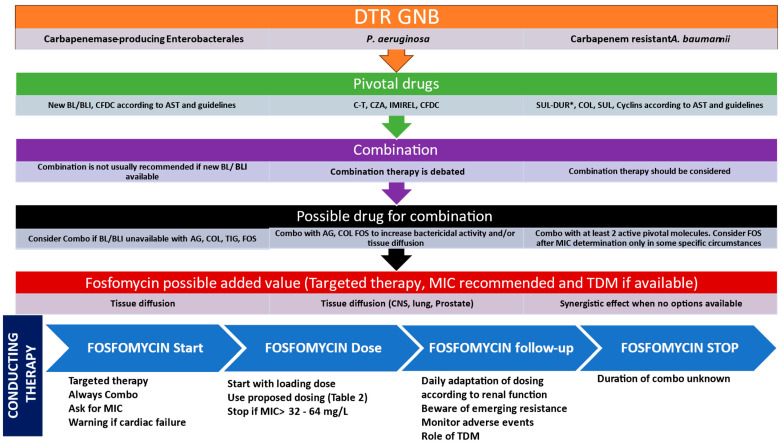
**Suggested treatment of severe DTR-GNB in ICU patients and suggested positioning of Fosfomycin use.** AST: antimicrobial susceptibility testing, C-T: ceftolozane-tazobactam, BL/BLI: beta-lactam/betalactamase inhibitor; CFDC: cefiderocol; CZA: ceftazidime avibactam; IMIREL: imipenem-relebactam; SUL: Sulbactam; SUL-DUR: sulbactam-durlobactam; AG: aminoglycosides, TIG: glycylcyclins, COL: colistin; FOS: Fosfomycin, AST: antibiotic susceptibility testing; TDM: therapeutic drug monitoring; CNS: central nervous system; MIC: minimum inhibitory concentration.

Pending further high-quality studies, we believe that IV fosfomycin—always as part of combination therapy—should be considered in selected clinical scenarios involving critically ill patients with DTR-GNB infections. Its proposed positioning is summarized in the accompanying figure (Figure 2). Future randomized controlled trials and cohort studies are eagerly awaited to better define its indications.

## 12. Conclusions and Future Directions

IV fosfomycin offers a valuable, cost-effective salvage option for severe DTR Gram-negative infections, especially when used in combination with other active agents. Its unique mechanism and broad in vitro activity make it an attractive candidate for “last-resort” therapy. However, the absence of high-quality clinical trials, variable susceptibility patterns, high dosing requirements, and potential for resistance emergence limit its broader adoption.

In practice, IV fosfomycin should be reserved for carefully selected patients, with rigorous microbiological surveillance and adherence to stewardship principles. Further prospective studies and clearer PK/PD guidance are needed to cement its place in the therapeutic armamentarium against DTR Gram-negative pathogens.

## Figures and Tables

**Table 1:** Potential in vitro activity of fosfomycin against carbapenem-resistant Gram-negative bacilli adapted from [41].

Multi or Carbapenem-Resistant GNB *	In Vitro Activity of Fosfomycin
ESBLs	YES
CRE non-CP	+/−
CRE-KPC	+/−
CRE-OXA-48	+/−
CRE-MBL	+/−
CRPA non-MBL	+/−
CRAB	NO **

CRAB: carbapenem-resistant *Acinetobacter baumannii*; CRE: carbapenem-resistant Enterobacterales; CRE non-CP: non-carbapenemase producing CRE; CRE-KPC: CRE *Klebsiella pneumoniae* carbapenemase producing CRE; CRE-OXA-48: OXA-48 producing CRE; CRE-MBL: metallo-beta-lactamases producing CRE; CRPA non-MBL: non-metallo-beta-lactamase producing carbapenem-resistant *Pseudomonas aeruginosa*; ESBLs: extended-spectrum beta-lactamases. (*) *Morganella morganni* is naturally resistant to fosfomycin. (**) Fosfomycin association with a pivotal antibiotic is suggested by cohort studies (see text).

**Table 2:** Dosing of IV fosfomycin and dosing adjustments in renal impairment and RRT adapted from [48,56,60].

FDA-approved prescribing dosage of IV fosfomycin [48]	EMA-approved prescribing dosage of IV fosfomycin [60]	Dosing suggestions ^§^ based on the population PK study by Götz et al. for bactericidal activity [56]			
Loading dose (1st dose) 6 g	Loading dose (1st dose) First dose should be increased by 100% but must not exceed 8 g	MIC 32 mg/L		MIC 64 mg/L	
Maintenance dose based on eCrCl	Maintenance dose based on CrCl (CrClpatient/CrClnormal)	eGFR_MDRD_ mL/min/1.73 m^2^ and CRRT ^~^ or PIRRT ^~^	eGFR_MDRD_ mL/min/1.73 m^2^ and No CRRT or PIRRT	eGFR_MDRD_ mL/min/1.73 m^2^ and CRRT ^~^ or PIRRT ^~^	eGFR_MDRD_ mL/min/1.73 m^2^ and No CRRT or PIRRT
>50: 6 g/8 h	≥80: 12–24 g divided in 2–3 doses (dose not >8 g)		91–120: 8 g/8 h ^±^		91–120: N/A
41–50: 4 g/8 h	40 (0.333): 70% * (in 2–3 doses)	61–90: 8 g/8 h	61–90: 8 g/8 h	61–90: N/A	61–90: N/A
31–40: 3 g/8 h	30 (0.250): 60% * (in 2–3 doses)	31–60: 8 g/8 h	31–60: 5 g/8 h	31–60: N/A	31–60: 8 g/8 h ^±^
21–30: 5 g/8 h	20 (0.167): 40% * (in 2–3 doses)	≤30: 5 g/8 h	≤30: 4 g/8 h	≤30: 8 g/8 h ^±^	≤30: 8 g/8 h
11–20: 3 g/24 h	10 (0.083): 20% * (in 1–2 doses)	0 (anuria): 4 g/8 h		0 (anuria): 8 g/8 h	
**Hemodialysis (HD)** On HD days, dose to be given after the end of each HD session (cleared during HD by 60–80%)	**Hemodialysis (HD)** Patients on chronic intermittent HD (every 48 h): 2 g at the end of each HD session				
	**Post-dilution VVHF** No adjustment needed (effectively eliminated)				

CRRT: continuous renal replacement therapy; eCrCl: estimated creatinine clearance; eGFR_MDRD_: estimated glomerular filtration rate in ml/min/1.73 m^2^ calculated using the Modification of Diet in Renal Disease equation; h: hour; MIC: minimum inhibitory concentration; N/A: not available; PIRRT: prolonged intermittent renal replacement therapy; RRT: renal replacement therapy; VVHF: veno-venous hemofiltration; * dose is expressed as % of appropriate dose if renal function was normal; ^§^ the recommended dosages represent the lowest dosage achieving probability of target attainment (PTA) ≥ 90%, apart from those with the symbol ^±^ that PTA achieved was ≥80%; ^~^ CRRT dialysate flow rate 42 mL/min and PIRT dialysate flow rate 250 mL/min.

**Table 3:** Summary of recommendations of ESCMID guidelines for the treatment of MDR GNB, which mention fosfomycin, adapted from [41].

Recommendation	Strength of Recommendation	Level of Evidence
For cUTI in patients without septic shock, IV fosfomycin is recommended or, conditionally, aminoglycosides when active in vitro for short durations of therapy.	Strong for fosfomycin Conditional for aminoglycosides	High for fosfomycin Moderate for aminoglycosides
For CRE, there is no evidence to recommend for or against the use of imipenem-relebactam and IV fosfomycin monotherapies (at the time of writing of the guidelines).	No recommendation	
For the treatment of severe infections caused by CRE with in vitro susceptibility only to polymyxins, aminoglycosides, tigecycline, or fosfomycin or in case of unavailability of BL/BLI, use of two in vitro active drugs is suggested, without any recommendation for or against a specific combination.	Conditional	Moderate
For the treatment of severe CRPA infections with polymyxins, aminoglycosides, or fosfomycin, use of two in vitro active drugs is suggested, without any recommendation for or against a specific combination.	Conditional	Very low

BL/BLI: beta-lactam/beta-lactamase inhibitor; CRE: carbapenem-resistant Enterobacterales; CRPA: carbapenem-resistant *Pseudomonas aeruginosa*; cUTI: complicated urinary tract infection; IV: intravenous.

## Data Availability

No new data were created or analyzed in this study.

## Abbreviations

The following abbreviations are used in this manuscript:

AMR	Antimicrobial Resistance
APACHE II score	Acute Physiology and Chronic Health Evaluation II
AUC	Area Under the Concentration–Time Curve
AUC_24–48_	Area Under the Concentration–Time Curve for 24 to 48 h
AUROC	Area Under the Receiver Operating Characteristic Curve
*bla*	Beta-Lactamase Gene
BL/BLI	Beta-Lactam/Beta-Lactamase Inhibitor
cUTIs	Complicated Urinary Tract Infections
CLSI	Clinical and Laboratory Standards Institute
Cmin	Minimum Concentration
CNS	Central Nervous System
CPE	Carbapenemase-Producing Enterobacterales
CRE	Carbapenem-Resistant Enterobacterales
Css	Steady State Concentration
CrCl	Creatinine Clearance
CRAB	Carbapenem-Resistant *Acinetobacter baumannii*
CRRT	Continuous Renal Replacement Therapy
DTR	Difficult-to-Treat Resistance
eGFR	Estimated Glomerular Filtration Rate
ESCMID	European Society of Clinical Microbiology and Infectious Diseases
EMA	European Medicines Agency
ESBLs	Extended-Spectrum Beta-Lactamases
EUCAST	European Committee on Antimicrobial Susceptibility Testing
FDA	Food and Drug Administration (United States)
GNB	Gram-Negative Bacilli
HAP	Hospital-Acquired Pneumonia
HR	Hazard Ratio
ICU	Intensive Care Unit
IDSA	The Infectious Diseases Society of America (IDSA)
IPTW	Inverse-Probability-of-Treatment Weighting
IQR	Interquartile Range
MIC	Minimum Inhibitory Concentration
MDR	Multidrug-Resistant
MDRD	Modification of Diet in Renal Disease
MRSA	Methicillin-Resistant *Staphylococcus aureus*
MurA	UDP-N-acetylglucosamine Enolpyruvyl Transferase
NDM	New Delhi Metallo-β-Lactamase
PK/PD	Pharmacokinetics/Pharmacodynamics
PIRRT	Prolonged Intermittent Renal Replacement Therapy
PTA	Probability of Target Attainment
RCT	Randomized Controlled Trial
SOFA score	Sequential Organ Failure Assessment
T	Time
TDM	Therapeutic Drug Monitoring
US	United States of America
VAP	Ventilator-Associated Pneumonia
VRE	Vancomycin-Resistant Enterococci
vs.	Versus
XDR	Extensively Drug-Resistant
